# Femoral extra‐physeal ACL reconstruction in skeletally immature patients shows a low rate of clinically relevant growth disturbances: A systematic review

**DOI:** 10.1002/jeo2.70849

**Published:** 2026-07-16

**Authors:** Lorenzo Bini, Tosca Cerasoli, Claudio Rossi, Giulio Maria Marcheggiani Muccioli, Stefano Zaffagnini, Alberto Grassi

**Affiliations:** ^1^ Department of Biomedical and Neuromotor Sciences (DIBINEM) University of Bologna Bologna Italy; ^2^ II Orthopaedic and Traumatologic Clinic IRCCS Istituto Ortopedico Rizzoli Bologna Italy

**Keywords:** anterior cruciate ligament, growth disturbances, paediatric, physeal‐sparing, skeletally immature

## Abstract

**Purpose:**

Physeal‐sparing techniques aim to reduce growth disturbances after anterior cruciate ligament reconstruction (ACLR) in skeletally immature patients, but evidence on femoral extra‐physeal approaches remains limited. The aim of this study was to systematically review the literature on femoral extra‐physeal ACLR in patients with open physes and to evaluate the reported incidence and severity of growth disturbances, including potential influencing factors, such as surgical technique and lateral extra‐articular tenodesis.

**Methods:**

A systematic search was conducted according to Preferred Reporting Items for Systematic Reviews and Meta‐Analyses guidelines. Studies including skeletally immature patients undergoing ACL reconstruction with femoral extra‐physeal techniques were included. Growth disturbances were defined as limb length discrepancy or angular deformity. Graft failure was defined as graft rupture, revision surgery or recurrent instability.

**Results:**

A total of 579 patients were included with a mean age of 12 years. The over‐the‐top technique was used in 176 patients, the Kocher–Micheli technique in 327 and the Clocheville technique in 76. Growth disturbances were reported in 26 patients (4.5%); overgrowth was the most frequent pattern. Most of the growth disturbances were minor (3.8%). No patient required surgical correction. Graft failure occurred in 30 patients (5.2%).

**Conclusion:**

Paediatric ACL reconstruction using femoral extra‐physeal techniques was associated with a low rate of clinically relevant growth disturbances. Most deformities were mild, and no corrective procedure was required. The graft failure rate was also low. These findings should be interpreted with caution given the heterogeneity of the included studies.

**Level of Evidence:**

Level IV.

## Graphical abstract

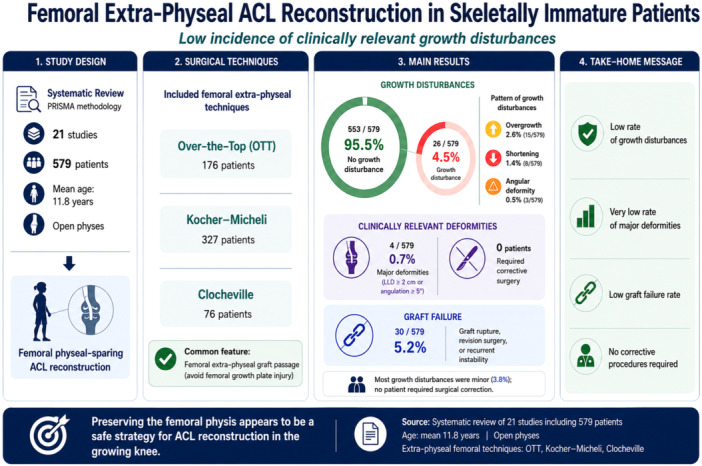

**Abbreviations**
ACL: anterior cruciate ligament
ACLR: anterior cruciate ligament reconstruction
GR: gracilis
ITB: ileo‐tibial band
LET: lateral extra‐articular tenodesis
LLD: leg length discrepancy
OTT: over the top
RTS: return to sport
ST: semitendinosus

## INTRODUCTION

The incidence of anterior cruciate ligament (ACL) rupture in paediatric patients has drastically increased in the last 20 years and is expected to rise further [3, 13]. The importance of an early ACL reconstruction (ACLR) is well documented as it reduces instability, the risk of articular cartilage and meniscal lesions, leading to better outcomes in terms of return to sport (RTS) [3, 12, 43].

**Figure 1:**
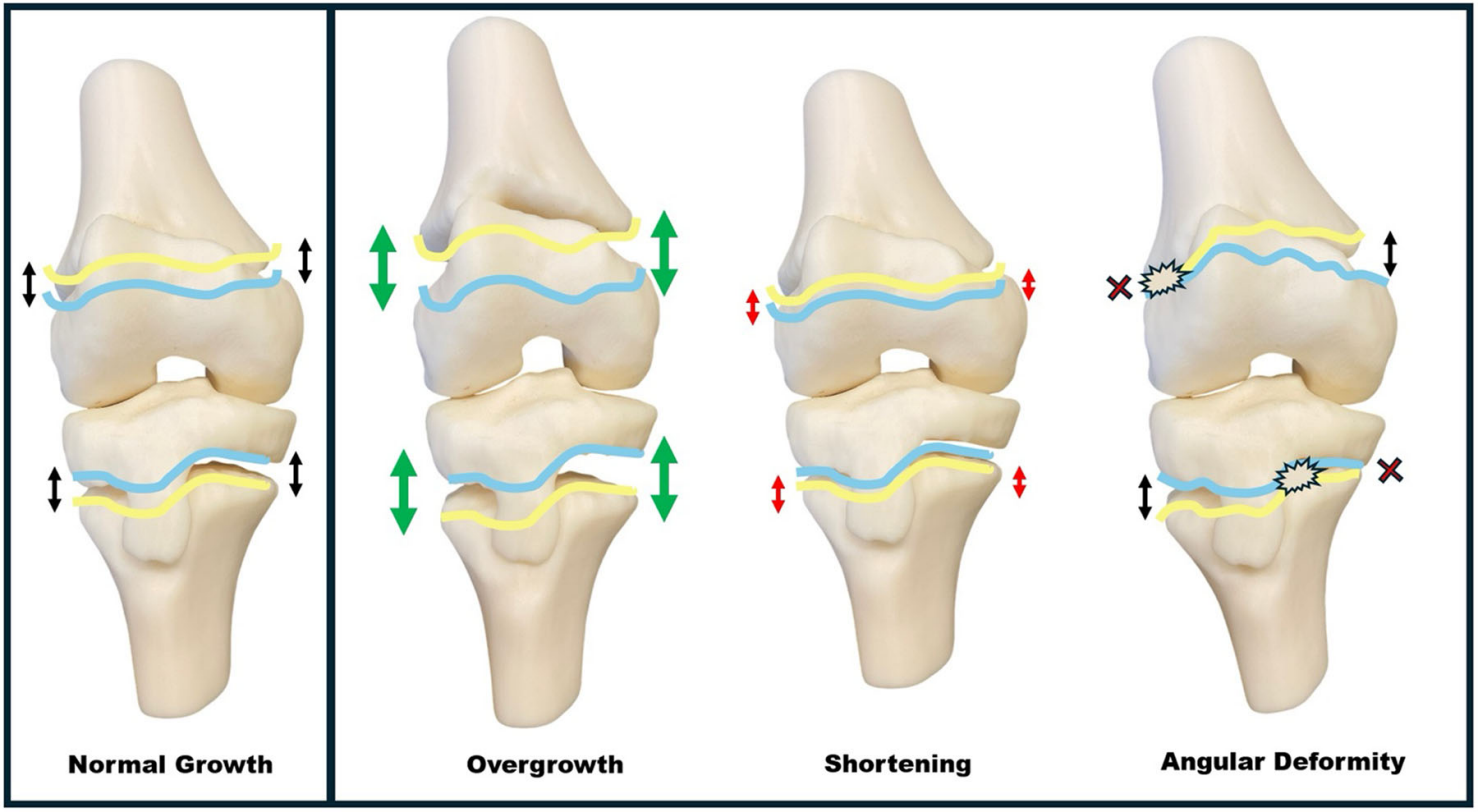
Mechanism of growth plate development disturbances.

Despite growing evidence supporting surgical management, ACLR in paediatric patients is still approached with caution because of physeal injury risk, which may lead to growth disturbances such as overgrowth, growth arrest or angular deformities (Figure 1) [8]. These alterations may result from physeal injury or local biological stimulation following surgery [28]. Preserving growth plate integrity is therefore a key aim in paediatric ACLR, with the femoral physis being of particular concern given its predominant contribution to lower‐limb growth [42]. In order to avoid trans‐physeal drilling, various techniques have been proposed, including partial trans‐physeal procedures, where only one tunnel is drilled through the physes (typically the tibial one) [7], and physeal‐sparing techniques, which avoid violating either growth plate by drilling both tunnels within the respective epiphyses (all‐epiphyseal) [1] or by avoiding any osseous tunnel altogether (extra‐physeal) [19].

Several physeal‐sparing techniques have been described: In 1999, Robert et al. described the Clocheville technique [35], featuring an extra‐physeal femoral tunnel and an all‐epiphyseal tibial tunnel, using the patellar tendon as a graft. In 2006, Kocher and Micheli described a completely extra‐physeal approach where a strip of iliotibial band (ITB), left attached at its distal insertion, is routed in the over‐the‐top (OTT) position, passed through the notch and beneath the intermeniscal ligament, and finally sutured to the anterior tibial periosteum [19], providing an intra‐articular ACLR with a lateral extra‐articular tenodesis (LET). In 2019, Roberti di Sarsina et al. [36] described the results of a modified ‘OTT and LET’ procedure, originally described for adult patients by Marcacci et al. [26], using a double‐strand gracilis and semitendinosus graft; this technique combines an extra‐physeal OTT femoral passage with an epiphyseal tibial tunnel, followed by a concomitant LET. These three techniques share the same guiding principle of femoral extra‐physeal fixation, which has shown overall good clinical results [33].

Previous systematic reviews have evaluated growth disturbances following paediatric ACLR, but no study has specifically addressed femoral extra‐physeal techniques, which could be considered among the first options in young patients [41]. The aim of the present study was to systematically review the literature on femoral extra‐physeal ACLR in patients with open physes, in order to evaluate the reported incidence and severity of postoperative growth disturbances and potential influencing factors, including surgical technique, tibial tunnel management and the use of LET. We hypothesized that femoral extra‐physeal ACLR is associated with a low rate of growth disturbances, that trans‐physeal drilling could be linked to a higher risk of disturbances, and that the addition of LET may not be associated with a higher rate of deformities.

## METHODS

**Figure 2:**
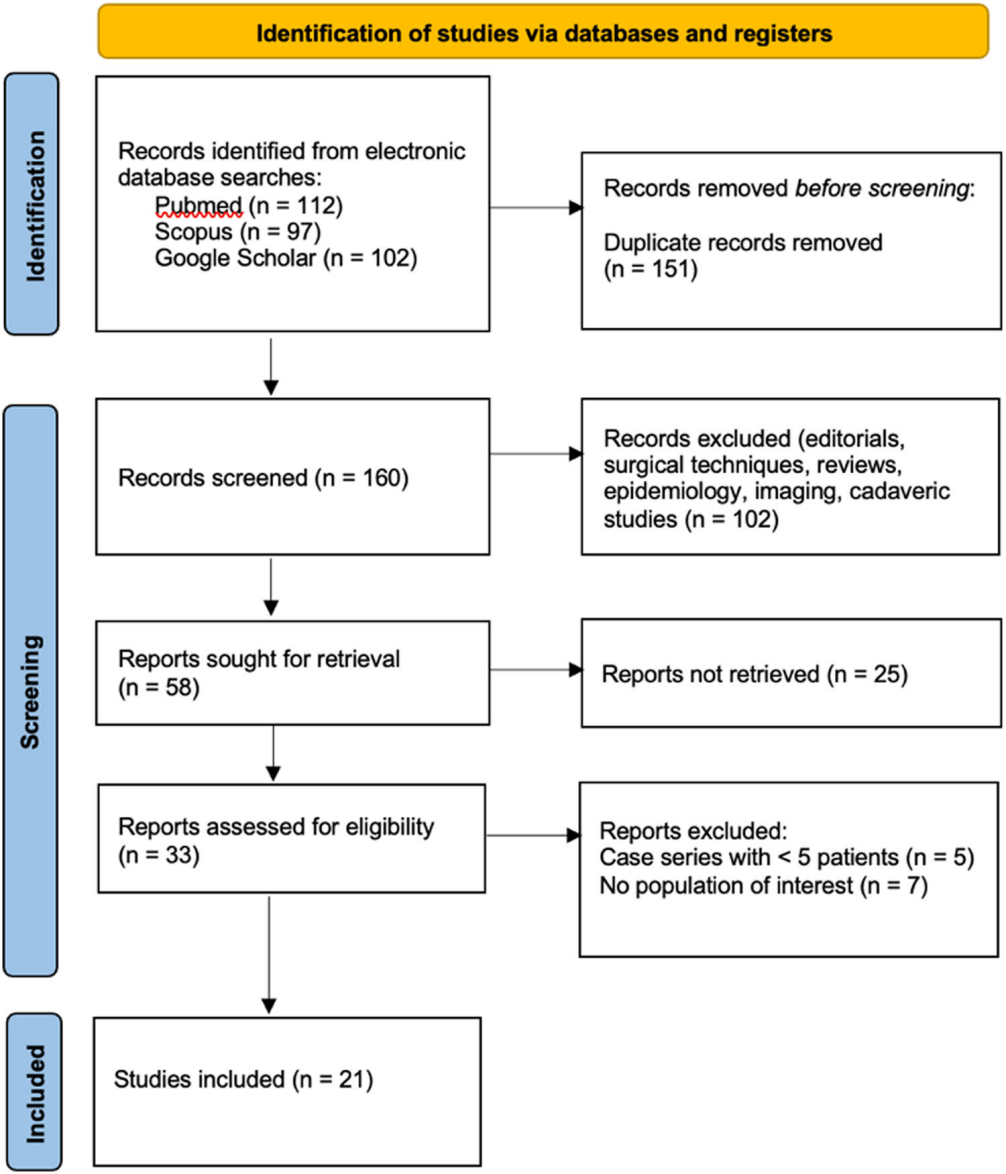
Study selection according to Preferred Reporting Items for Systematic Reviews and Meta‐Analyses (PRISMA) guidelines.

We conducted a systematic review according to Preferred Reporting Items for Systematic Reviews and Meta‐Analyses (PRISMA) guidelines [31]. The protocol is registered in the PROSPERO database (CRD420251181578). A comprehensive search was performed in PubMed, Scopus and Google Scholar ab initio to November 2025. The following search string was used in PubMed and adapted for the other databases: (‘anterior cruciate ligament’ OR ‘ACL’) AND (‘over‐the‐top’ OR ‘OTT’ OR ‘extra‐physeal’ OR ‘physeal‐sparing’) AND (‘paediatric’ OR ‘immature’ OR ‘prepubescent’) AND (‘physes’ OR ‘physis’). In addition, the reference lists of included studies were manually screened to identify potentially relevant articles. Every article found was checked by two independent authors. Studies were included if they described primary isolated ACLR performed with a femoral extra‐physeal approach in skeletally immature patients with open physes, clearly reporting the surgical technique and graft type and providing postoperative data on complications, specifically growth disturbances (limb length discrepancy or angular deformity) and graft failures. Skeletal immaturity was defined by the presence of open physes on pre‐operative imaging as reported by the authors of each included study, not based on chronological age. Studies were excluded if they were case reports including fewer than five patients, as well as cadaveric, animal or biomechanical studies. Studies not available in English were excluded. Studies not retrievable despite attempts through institutional access and contact with authors were also excluded (Figure 2). When authors were not in agreement on whether to include an article, a third senior author was consulted to reach a final decision.

The methodological quality of the included studies was assessed using the Downs and Black checklist for risk of bias assessment [11]. The checklist assigns to studies an excellent ranking for scores ≥26, good for scores from 20 to 25, fair for scores between 15 and 19 and poor for scores ≤14 points. Data on the number of patients included in each study, mean age, sex and graft type were collected. The surgical technique was analysed, categorizing the tibial side as trans‐physeal, extra‐physeal or no tunnel, while the femoral side was consistently extra‐physeal. Any additional data available were collected, including the type of fixation used, if any LET was performed, and the number of graft failures reported. Graft failure was defined as graft rupture, need for revision ACLR or persistence of subjective or objective instability after surgery requiring surgical intervention, defined as recurrent symptomatic giving‐way episodes and/or objective anterior or rotational knee laxity confirmed on clinical examination. Every article was checked systematically for data regarding growth disturbances, including whether preoperative deformities were assessed and how postoperative changes were evaluated (radiological and/or clinical follow‐up). Any leg length discrepancy (LLD) observed during follow‐up was recorded and classified as minor (LLD between 1 and 10 mm) and major (LLD greater than 10 mm) according to the criteria adopted in one of the most recent meta‐analyses [33]. Similarly, any coronal angular deformity detected at follow‐up was reported and categorized as minor (varus or valgus deviation between 1° and 3° compared to the contralateral) and major (any deviation greater than 3° compared to the contralateral) [33]. Finally, we reported if any surgical correction of the deformity was needed. Due to the significant heterogeneity among the included studies in terms of study design, surgical techniques, outcome definitions, follow‐up duration and methods used to assess growth disturbances, a quantitative meta‐analysis was not considered appropriate. Therefore, a descriptive analysis of the available data was performed. Data were summarized using descriptive statistics, including means, ranges and proportions where appropriate.

## RESULTS

A total of 21 studies, published between 1991 and 2025, met the inclusion criteria, reporting 579 cases of ACLR performed in skeletally immature patients using a femoral extra‐physeal passage. Overall, the majority of patients were male (71.3%), and the mean age at surgery was 11.8 years (range, 8–16 years). A total of 13 studies (176 patients) used an OTT technique with different types of graft (mainly hamstring tendons), five studies (327 patients) reported the results of the Kocher–Micheli technique using the ITB as a graft, and finally three studies (76 patients) used the Clocheville technique with the patellar tendon as graft (Table 1). The mean reported follow‐up was 45.4 ± 33.5 months.

**Table 1:** Surgical techniques and details of every study.

Article	Year	Patients (no.)	Mean age (years)	Femoral passage	Femoral fixation	Tibial passage	Tibial fixation	Graft type	Lateral tenodesis	Surgical failures
Gomez‐Caceres et al. [15]	2024	12	13.2	Extra‐physeal (OTT)	Screw	Epiphyseal	No	GR + ST	Yes (modified Lemaire)	0
Kamei et al. [18]	2022	10	12.1 (11–13)	Extra‐physeal (OTT)	Staples	Trans‐physeal	Staples	ST	No	0
Shamrock et al. [40]	2022	12	12.8 (10–16)	Extra‐physeal (OTT)	Screw	Trans‐physeal	Screw	GR + ST	No	2 (16.7%)
Nagai et al. [29]	2019	14	14	Extra‐physeal (OTT)	Screw	Trans‐physeal	Screw	N.R.	No	2 (14.3%)
Demange et al. [10]	2014	12	10.7 (8–12)	Extra‐physeal (OTT)	Screw	Trans‐physeal	Screw	ST	No	3 (25%)
Bisson et al. [4]	1998	9	13 (10–15)	Extra‐physeal (OTT)	Staples or screw	Trans‐physeal	No	GR + ST	No	2 (22.2%)
Lo et al. [24]	1997	5	12.9 (8–14)	Extra‐physeal (OTT)	Staples	Trans‐physeal	No	2 ST 1 GR + ST 2 QUAD	No	0
Janarv Per‐Mats et al. [17]	1996	20	13.5 (10–15)	Extra‐physeal (OTT)	Staples	Epiphyseal	No	12 ST 4 PT 4 N.R.	No	1 (5%)
Parker et al. [32]	1994	6	13.3 (10–14)	Extra‐physeal (OTT)	Staples	Extra‐physeal (no tunnel)	Staples	5 GR + ST 1 ST	No	0
Andrews et al. [2]	1994	8	13.5 (10–15)	Extra‐physeal (OTT)	Staples	Trans‐physeal	Staples	5 ITB 3 Achilles allograft	No	1 (12.5%)
Brief et al. [6]	1991	6	14.8 (14–16)	Extra‐physeal (OTT)	Staples	Extra‐physeal (no tunnel)	No	GR + ST	Yes (modified Lemaire)	0
Lanzetti et al. [22]	2020	42	12.5 (11–14)	Extra‐physeal (OTT)	Staples	Epiphyseal	No	GR + ST	Yes (Marcacci)	2 (4.8%)
Roberti di Sarsina et al. [36]	2018	20	12.3 (8–13)	Extra‐physeal (OTT)	Staples	Epiphyseal	No	GR + ST	Yes (Marcacci)	0
Lopez Martinez et al. [25]	2025	19	10 (7–12)	Extra‐physeal (KM)	Sutures	Extra‐physeal (no tunnel)	Bone harpoon	ITB	Yes	0
Kocher et al. [20]	2018	237	11.2	Extra‐physeal (KM)	Sutures	Extra‐physeal (no tunnel)	Sutures	ITB	Yes	9 (3.8%)
Willimon et al. [44]	2015	22	11.8 (9–14)	Extra‐physeal (KM)	Sutures	Extra‐physeal (no tunnel)	Sutures	ITB	Yes	3 (13.6%)
Kocher et al. [19]	2006	44	10.3	Extra‐physeal (KM)	Sutures	Extra‐physeal (no tunnel)	Sutures	ITB	Yes	2 (4.5%)
Nakhostine et al. [30]	1995	5	14 (12–15)	Extra‐physeal (KM)	N.R.	Epiphyseal	Staples	ITB	Yes	0
Severyns et al. [39]	2016	11	13.5 (11–16)	Extra‐physeal (Clocheville)	Screw	Extra‐physeal (no tunnel)	Staples	PT	No	0
Bonnard et al. [5]	2011	57	12.2 (6–14)	Extra‐physeal (Clocheville)	Screw	Extra‐physeal (no tunnel)	Sutures	PT	No	3 (5.3%)
Robert et al. [35]	1999	8	11.4 (9–13)	Extra‐physeal (Clocheville)	Screw	Extra‐physeal (no tunnel)	Staples	PT	No	0

Abbreviations: GR, gracilis; ITB, iliotibial band; KM, Kocher–Micheli; N.R., not reported; OTT, over the top; PT, patellar tendon; QUAD, quadricipital tendon; ST, semitendinosus.

### Growth disturbance assessment

Postoperative deformities were assessed radiographically in 13 studies [2, 5, 10, 18, 19, 22, 24, 30, 32, 35, 36, 39, 44] including 250 patients (43.2%) and clinically in two studies [4, 17] including 29 patients (5.0%). In the remaining studies [6, 15, 20, 25, 29, 40] (300 patients, 51.8%), the method for growth disturbances assessment was not specifically reported. Only three studies [5, 18, 22] (109 patients, 18.8%) performed a pre‐operative radiographic evaluation.

### Incidence of growth disturbances

Among the 579 ACLRs included, 26 patients (4.5%) presented postoperative growth disturbances, most of which were minor (22 cases, 3.8%). Only four patients (0.7%) developed major deformities (Table 2). The most frequent pattern was overgrowth (15/579 patients, 2.6%), followed by shortening (8/579 patients, 1.4%) and angular deformity (3/579 patients, 0.5%). Only 1/579 patient (0.2%) was reported to have a mixed deformity of varus and overgrowth. None of the reported deformities required corrective surgery (Table 2). As all included techniques avoid violation of the femoral physis, the reported growth disturbances are unlikely to originate from the femur and are more plausibly related to tibial involvement or indirect biomechanical mechanisms, although their anatomical origin is not consistently specified across the included studies.

**Table 2:** Summary of growth disturbances reported and follow‐up duration.

Article	Patients (no.)	Femoral passage	Pre‐op evaluation	Post‐op evaluation	Total disturbances (%)	Major	Minor	Type	Surgical correction	Mean follow‐up (months)
Gomez‐Caceres et al. [15]	12	Extra‐physeal (OTT)	N.R.	N.R.	0 (0%)	0 (0%)	0 (0%)	—	0 (0%)	26.0
Kamei et al. [18]	10	Extra‐physeal (OTT)	Radiological	Radiological	0 (0%)	0 (0%)	0 (0%)	—	0 (0%)	32.4
Shamrock et al. [40]	12	Extra‐physeal (OTT)	N.R.	N.R.	0 (0%)	0 (0%)	0 (0%)	—	0 (0%)	27.6
Nagai et al. [29]	14	Extra‐physeal (OTT)	N.R.	N.R.	0 (0%)	0 (0%)	0 (0%)	—	0 (0%)	26.4
Demange et al. [10]	12	Extra‐physeal (OTT)	N.R.	Radiological and clinical	0 (0%)	0 (0%)	0 (0%)	—	0 (0%)	220.0
Bisson et al. [4]	9	Extra‐physeal (OTT)	N.R.	Clinical	3 (33%)	0 (0%)	3 (33%)	3 Shortening	0 (0%)	39.0
Lo et al. [24]	5	Extra‐physeal (OTT)	N.R.	Radiological	3 (60%)	0 (0%)	3 (60%)	2 Shortening 1 Overgrowth	0 (0%)	88.8
Janarv Per‐Mats et al. [17]	20	Extra‐physeal (OTT)	N.R.	Clinical	0 (0%)	0 (0%)	0 (0%)	—	0 (0%)	36.0
Parker et al. [32]	6	Extra‐physeal (OTT)	N.R.	Radiological	5 (83.3%)	0 (0%)	5 (83.3%)	5 Overgrowth	0 (0%)	33.2
Andrews et al. [2]	8	Extra‐physeal (OTT)	N.R.	Radiological	5 (63%)	1 (12.5%)	4 (50%)	3 Shortening 2 Overgrowth	0 (0%)	58.0
Brief et al. [6]	6	Extra‐physeal (OTT)	N.R.	N.R.	0 (0%)	0 (0%)	0 (0%)	—	0 (0%)	36.0
Lanzetti et al. [22]	42	Extra‐physeal (OTT)	Radiological	Radiological	2 (4.8%)	1 (2.4%)	1 (2.4%)	2 Angular (Valgus)	0 (0%)	96.1
Roberti di Sarsina et al. [36]	20	Extra‐physeal (OTT)	N.R.	Radiological	3 (15%)	1 (5%)	2 (10%)	1 Overgrowth + Angular (Varus), 1 Angular (Varus) 1 Overgrowth	0 (0%)	54.0
Lopez Martinez et al. [25]	19	Extra‐physeal (KM)	N.R.	N.R.	0 (0%)	0 (0%)	0 (0%)	—	0 (0%)	24.0
Kocher et al. [20]	237	Extra‐physeal (KM)	N.R.	N.R.	0 (0%)	0 (0%)	0 (0%)	—	0 (0%)	25.8
Willimon et al. [44]	22	Extra‐physeal (KM)	N.R.	Radiological	0 (0%)	0 (0%)	0 (0%)	—	0 (0%)	36.0
Kocher et al. [19]	44	Extra‐physeal (KM)	N.R.	Radiological and clinical	0 (0%)	0 (0%)	0 (0%)	—	0 (0%)	63.6
Nakhostine et al. [30]	5	Extra‐physeal (KM)	N.R.	Radiological	1 (20%)	1 (20%)	0 (0%)	1 Overgrowth	0 (0%)	52.8
Severyns et al. [39]	11	Extra‐physeal (Clocheville)	N.R.	Radiological	0 (0%)	0 (0%)	0 (0%)	—	0 (0%)	25.2
Bonnard et al. [5]	57	Extra‐physeal (Clocheville)	Radiological and clinical	Radiological	0 (0%)	0 (0%)	0 (0%)	—	0 (0%)	66.0
Robert et al. [35]	8	Extra‐physeal (Clocheville)	N.R.	Radiological	4 (50%)	0 (0%)	4 (50%)	4 Overgrowth	0 (0%)	42.0

Abbreviations: KM, Kocher–Micheli; N.R., not reported; OTT, over the top.

### Subgroup analysis

#### Surgical technique

Among the 176 patients that underwent ACLR with an OTT technique, 21 patients (11.9%) presented growth disturbance; of which 3 (1.7%) were major. Most disturbances were minor and included both overgrowth (42.9%) and shortening (38.1%) patterns.

Among the 327 patients that underwent ACLR with the Kocher–Micheli technique, only 1 out of 327 patients (0.3%) presented a growth disturbance. In the Clocheville group, 4 out of 76 patients (5.2%) presented minor deformities. Detailed distributions are reported in Table 3.

**Table 3:** Growth disturbances sorted by femoral passage, tibial passage and LET.

	Total patients	Growth disturbances (% of total patients)	Major (%)	Minor (%)
Surgical technique				
OTT	176	21 (11.9%)	3 (1.7%)	18 (10.2%)
KM	327	1 (0.3%)	1 (0.3%)	0 (0%)
Clocheville	76	4 (5.3%)	0 (0%)	4 (5.3%)
Tibial passage				
Trans‐physeal	70	11 (15.7%)	1 (1.4%)	10 (14.3%)
Epiphyseal	99	6 (6.1%)	3 (3%)	3 (3%)
Extra‐physeal‐no tunnel	410	9 (2.2%)	0 (0%)	9 (2.2%)
LET				
Yes	407	6 (1.5%)	3 (0.7%)	3 (0.7%)
No	172	20 (11.6%)	1 (0.6%)	19 (11%)

Abbreviations: KM, Kocher–Micheli; LET, lateral extra‐articular tenodesis; OTT, over the top.

#### Tibial passage

The tibial passage varied among trans‐physeal (70 patients), epiphyseal (99 patients) and extra‐physeal (410 patients) techniques. Growth disturbances were reported across all groups, with major deformities ranging from 0% to 3% (Table 3).

#### Extra‐articular tenodesis

A total of 407 out of 579 patients (70.3%) underwent LET. When ACLR was combined with LET, 3 cases (0.7%) of major growth disturbances were observed, compared to 1 case (0.6%) without LET (Table 3).

### Complications and graft failures

No patients (0%) underwent reoperation due to minor or major growth disturbance or required surgical corrections. A total of 30 patients (5.2%) experienced a graft failure, ranging from 0% in 10 studies including either OTT, Kocher–Micheli and Clocheville technique [6, 15, 18, 24, 25, 30, 32, 36, 39] to 25% in one cohort with OTT technique with hamstrings, trans‐physeal tibial tunnel and no lateral tenodesis [10].

### Risk of bias and quality of evidence

**Figure 3:**
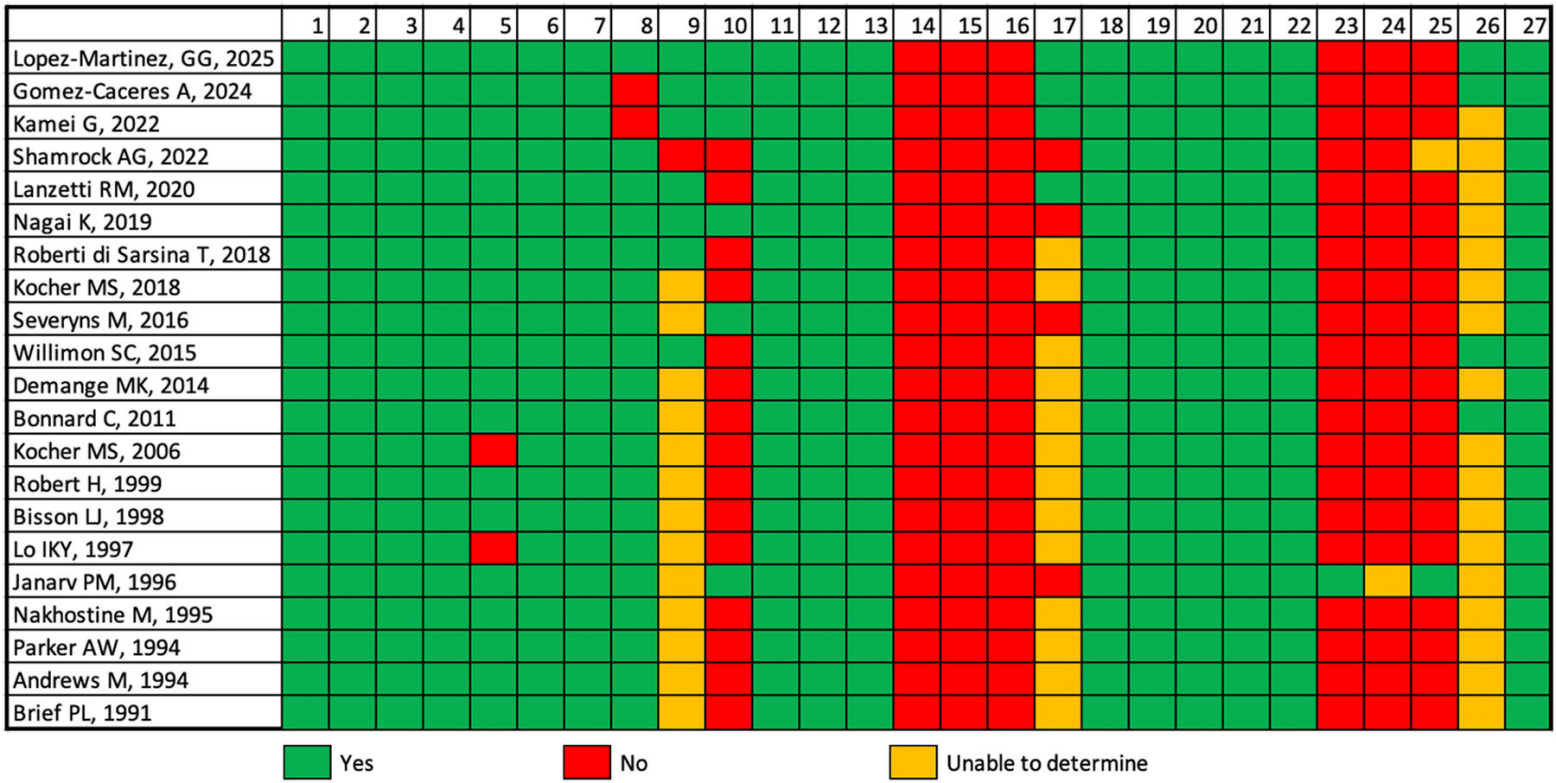
Downs and Black's checklist.

According to the Downs and Black checklist for risk of bias assessment, three studies were classified as good and 18 as fair [11] (Figure 3).

## DISCUSSION

The present systematic review suggested that femoral extra‐physeal ACLR in skeletally immature patients may represent a safe approach, with growth disturbances appearing uncommon (4.5%) and clinically relevant deformities occurring in only a minority of cases (0.7%). Graft failure rates were similarly low (5.2%), further supporting the possible safety of this technique. When compared with previously reported data on paediatric ACLR using trans‐physeal or all‐epiphyseal techniques [33], the incidence of growth disturbances observed was comparable, although direct comparisons are limited by differences in study design, patient selection and assessment methods.

As the femoral physis contributes predominantly to lower‐limb longitudinal growth, its preservation is of primary importance in skeletally immature patients undergoing ACLR. For this reason, the present review was conducted to specifically address the extra‐physeal femoral approach. Biomechanical investigations have demonstrated that the extra‐articular femoral OTT configuration ensures satisfactory anteroposterior and rotatory knee stability and may even reduce posterior femoral contact stresses compared to all‐epiphyseal reconstructions [23, 27].

Despite including different surgical techniques, all procedures shared a common principle of femoral extra‐physeal graft routing. A higher incidence of growth disturbances was observed in the OTT subgroup, likely reflecting more systematic postoperative assessment (9/13 authors reported a postoperative systematic check for growth abnormalities) rather than true technical differences. Only 3 patients (1.7%) in the OTT group developed a major deformity (LLD > 10 mm or angular deviation > 3°) when using the cut‐off generally adopted in the literature for paediatric patients [9, 33, 45]. This value is even stricter compared to LLD cut‐offs adopted for adult patients (20 mm).

On the other hand, in the biggest cohort included in the current review [20], accounting for almost 41% of the total patients (237 patients), the authors did not report any postoperative disturbance assessment. Patients were clinically evaluated at a minimum 2‐year follow‐up, which would have made it possible to detect major growth disturbances, but no radiological evaluation was performed. This lack of assessment probably contributed to the underreporting of growth disturbances. Another interesting finding was that most reported growth disturbances were minor, asymptomatic and diagnosed without comparison to preoperative imaging. Considering that physiological limb‐length discrepancies are relatively common in the general population [38], some differences may not be directly attributable to surgery.

Other technical variables were taken into consideration in the growth abnormalities assessment, such as the type of tibial passage and addition of LET. Although the tibial physis contribution to overall limb growth is lower than that of the femur [42], it remains vulnerable to asymmetric loading, tunnel malposition and excessive graft tensioning, which may predispose to angular deformities or graft elongation. Therefore, intra‐physeal, trans‐physeal or extra‐physeal tibial passage could be considered relevant for possible postoperative deformities. Despite a consistent femoral approach, several authors adopted a trans‐physeal approach, characterized by a physeal‐respecting technique which included a small tunnel diameter, perpendicular drilling through the centre of the tibial physis, avoidance of hardware or bone plugs across the growth plate and careful graft tensioning [34]. Using this approach, postoperative deformities were apparently uncommon, supporting the safety of the approach. These ‘physeal‐respecting’ concepts are supported by severe deformities described in case reports outside this review, such as the tibial deformity reported by Rozbruch et al. after ACLR with an Achilles allograft and a trans‐physeal tibial tunnel [37], or the 2.8 cm overgrowth reported by Zimmerman et al. following ACLR with a posterior tibialis allograft [46]. Severe deformities described in isolated reports likely reflect non‐physiological conditions rather than standard surgical practice.

The addition of LET, known to reduce graft failure rates [14, 16], did not increase the incidence of major growth disturbances in the included studies (0.7% vs. 0.6% without LET). Graft failure rate was also assessed in order to determine the efficacy of the included surgical procedures. The overall graft failure rate was low (5.2%), suggesting that satisfactory outcomes and graft survivorship are achievable even with non‐anatomical reconstructive techniques. However, a relevant variability in the failure rates was noted (from 0% to 20%) [4, 10]. Among the six authors reporting graft failure rates exceeding 10%, five did not perform a LET [2, 4, 10, 29, 40], suggesting the protective role of this procedure on graft survival [14, 21]. Notably, the addition of LET did not increase the incidence of growth disturbances, with comparable rates of major deformities between groups.

This systematic review presents several limitations. The interpretation of growth disturbances is significantly complicated by the absence of routine preoperative radiographic evaluation. Only a few studies assessed pre‐existing limb length discrepancy or angular deformity before surgery, despite evidence that minor asymmetries can be present physiologically [38]. Consequently, some of the postoperative differences reported may represent physiological variations rather than true surgical sequelae. Additionally, follow‐up duration was heterogeneous across all studies, limiting the interpretation of both growth disturbances and graft failure rates. Furthermore, the quality of the included studies was predominantly fair, with few studies rated as good, underscoring the need for prospective and comparative investigations with standardized imaging and clinical endpoints. Another limitation concerns the heterogeneity in sample size across the included series, ranging from small cohorts of five or six patients [6, 24, 30, 32] to large single‐centre studies including more than 200 reconstructions performed with the same technique [19]. In this single‐centre study, the authors did not perform post‐operative radiological assessment: this probably determined an underreporting of growth disturbances and should not be interpreted as a superiority of the Kocher–Micheli technique compared to the other extra‐physeal approaches described.

Finally, the studies span a wide time frame, reflecting the historical evolution of surgical techniques and introducing variability in both technique and reporting quality.

## CONCLUSIONS

Paediatric ACLR using extra‐physeal femoral techniques in skeletally immature patients was associated with a low reported incidence of clinically relevant growth disturbances, the vast majority of which were mild and asymptomatic. Major deformities were rare (0.7%), and no cases required corrective surgery. The overall graft failure rate was also low (5.2%).

While these findings suggested the use of femoral extra‐physeal techniques as a safe surgical option in the growing knee, they should be interpreted with caution given the heterogeneity and methodological limitations of the available evidence.

## AUTHOR CONTRIBUTIONS

*Conceptualization*: Alberto Grassi and Stefano Zaffagnini. *Methodology*: Alberto Grassi. *Validation*: Alberto Grassi and Stefano Zaffagnini. *Formal analysis*: Alberto Grassi and Lorenzo Bini. *Investigation*: Alberto Grassi, Lorenzo Bini, Claudio Rossi and Tosca Cerasoli. *Resources*: Alberto Grassi. *Data curation*: Alberto Grassi, Lorenzo Bini and Tosca Cerasoli. *Writing—original draft preparation*: Alberto Grassi, Lorenzo Bini and Tosca Cerasoli. *Writing—review and editing*: Giulio Maria Marcheggiani Muccioli, Lorenzo Bini and Tosca Cerasoli. *Supervision*: Alberto Grassi and Stefano Zaffagnini. *Project administration*: Alberto Grassi, Giulio Maria Marcheggiani Muccioli. All authors have read and agreed to the published version of the manuscript.

## FUNDING INFORMATION

The authors have no funding to report.

## CONFLICT OF INTEREST STATEMENT

The authors declare no conflict of interest.

## ETHICS STATEMENT

The authors have nothing to report.

## Data Availability

The data that support the findings of this study are openly available in the articles cited in the study.
